# No Serological Evidence of Influenza A H1N1pdm09 Virus Infection as a Contributing Factor in Childhood Narcolepsy after Pandemrix Vaccination Campaign in Finland

**DOI:** 10.1371/journal.pone.0068402

**Published:** 2013-08-08

**Authors:** Krister Melén, Markku Partinen, Janne Tynell, Maarit Sillanpää, Sari-Leena Himanen, Outi Saarenpää-Heikkilä, Christer Hublin, Päivi Olsen, Jorma Ilonen, Hanna Nohynek, Ritva Syrjänen, Terhi Kilpi, Arja Vuorela, Turkka Kirjavainen, Outi Vaarala, Ilkka Julkunen

**Affiliations:** 1 Department of Infectious Disease Surveillance and Control, National Institute for Health and Welfare, Helsinki, Finland; 2 Helsinki Sleep Clinic, Vitalmed Research Centre Helsinki and Haartman Institute, University of Helsinki, Helsinki, Finland; 3 University of Tampere and Department of Clinical Neurophysiology, Tampere University Hospital, Tampere, Finland; 4 Department of Pediatrics, Tampere University Hospital, Tampere, Finland; 5 Finnish Institute of Occupational Health, Helsinki, Finland; 6 Department of Child Neurology, Oulu University Hospital, Oulu, Finland; 7 Department of Clinical Microbiology, University of Eastern Finland, Kuopio, Finland; 8 Immunogenetics Laboratory, University of Turku, Turku, Finland; 9 Department of Vaccines and Immune Protection, National Institute for Health and Welfare, Helsinki, Finland; 10 Department of Pediatrics, Children’s Hospital, Helsinki University Hospital, Helsinki, Finland; 11 Department of Virology, University of Turku, Turku, Finland; INRS, Canada

## Abstract

**Background:**

Narcolepsy cataplexy syndrome, characterised by excessive daytime sleepiness and cataplexy, is strongly associated with a genetic marker, human leukocyte antigen (HLA) DQB1*06:02. A sudden increase in the incidence of childhood narcolepsy was observed after vaccination with AS03-adjuvanted Pandemrix influenza vaccine in Finland at the beginning of 2010. Here, we analysed whether the coinciding influenza A H1N1pdm pandemic contributed, together with the Pandemrix vaccination, to the increased incidence of childhood narcolepsy in 2010. The analysis was based on the presence or absence of antibody response against non-structural protein 1 (NS1) from H1N1pdm09 virus, which was not a component of Pandemrix vaccine.

**Methods:**

Non-structural (NS) 1 proteins from recombinant influenza A/Udorn/72 (H3N2) and influenza A/Finland/554/09 (H1N1pdm09) viruses were purified and used in Western blot analysis to determine specific antibody responses in human sera. The sera were obtained from 45 patients who fell ill with narcolepsy after vaccination with AS03-adjuvanted Pandemrix at the end of 2009, and from controls.

**Findings:**

Based on quantitative Western blot analysis, only two of the 45 (4.4%) Pandemrix-vaccinated narcoleptic patients showed specific antibody response against the NS1 protein from the H1N1pdm09 virus, indicating past infection with the H1N1pdm09 virus. Instead, paired serum samples from patients, who suffered from a laboratory confirmed H1N1pdm09 infection, showed high levels or diagnostic rises (96%) in H1N1pdm virus NS1-specific antibodies and very high cross-reactivity to H3N2 subtype influenza A virus NS1 protein.

**Conclusion:**

Based on our findings, it is unlikely that H1N1pdm09 virus infection contributed to a sudden increase in the incidence of childhood narcolepsy observed in Finland in 2010 after AS03-adjuvanted Pandemrix vaccination.

## Introduction

Narcolepsy with cataplexy is characterised by an excessive daytime sleepiness, cataplexy and disturbed nocturnal sleep. In addition, patients may have other rapid eye movement (REM) sleep-related symptoms such as hypnagogic hallucinations and sleep paralysis [1]. A deficit in the endogenous hypocretin (orexin) system due to neuronal destruction in the lateral hypothalamus is considered to be the primary pathophysiological mechanism of the disease in humans [2], [3], [4], [5]. Most often narcolepsy starts between 12–25 years of age, while onset before the age of 10 has been rare [6], [7]. The peak mode of onset age is around 15 years [8]. It is strongly associated with a genetic marker, human leukocyte antigen (HLA) DQB1*06:02, indicating an autoimmune-mediated process [9], [10]. Seasonality of the onset and association of the disease with upper respiratory tract infections, including H1N1 [11] and/or streptococcal infections [12], [13], suggest that infections could initiate or reactivate an immune response that leads to the loss of hypocretin secreting cells and narcolepsy in genetically susceptible individuals.

Previously, a 17-fold increase in the annual incidence of narcolepsy, as compared to previous years in children aged under 17, was observed in Finland in 2010 [14]. When vaccinated and unvaccinated children and adolescents were compared, about a 12-fold increase in the risk of narcolepsy was observed in vaccinated individuals [15]. It was considered likely that Pandemrix vaccination, perhaps together with other environmental factors, contributed to the increased incidence of narcolepsy in HLA DQB1*06:02-positive children in Finland in 2010 [14], [15]. More recently, an increased incidence of narcolepsy in Pandemrix-vaccinated children/adolescents was also observed in Sweden and U.K. [16], [17].

As the onset of narcolepsy has been shown to be seasonal, and the cases of narcolepsy increased following the 2009 H1N1 pandemic in China [11], we wanted to analyse whether the coinciding pandemic influenza A H1N1 infections contributed, together with the AS03-adjuvanted Pandemrix vaccination, to the sudden increase in the incidence of childhood narcolepsy that was observed in Finland in 2010.

## Methods

### Ethical Permissions

The study protocol was reviewed and approved by the Institutional Review Board of the National Institute for Health and Welfare (THL), Helsinki. Children with narcolepsy diagnosed after Pandemrix vaccination were recruited to the immunological narcolepsy study at the neurological outpatient clinics in Finnish children's hospitals during the years 2011 and 2012. The study protocol was approved by the Ethics Committee of participating hospitals and informed written consent was given both by children and their guardians.

The collection of serum samples from individuals suffering from an upper respiratory tract infection was approved by the Ethical Committee of the Pirkanmaa Hospital District (ETL-code numbers R09152M and R10075M). All patients gave their written informed consent for serum samples.

Control serum specimens for the study included serum samples collected in 2004/2005 prior to the 2009 influenza pandemic. The samples were made anonymous by destroying all person identification data before they were shipped to the National Institute for Health and Welfare (THL); only the age of the subjects and the sample collection dates were retained. Permission to use these anonymous serum specimens was based on ethical permissions given by the Helsinki-Uusimaa Health District Ethical committee: HUS 315/E0/05 (dated 6.9.2005; initially for polio and MMR antibody analyses) and 199/13/03/00/09 (dated on 30.6.2009; expanded for influenza antibody analyses). The studies fully comply with the rules concerning national and international regulations, privacy and data protection relevant to medical- and patient-oriented studies.

### Identification of Narcoleptic Patients

The present study comprised 45 narcoleptic patients aged 5 to 18 years at onset who fell ill in 2009 or 2010 after receiving Pandemrix vaccination in November or December 2009. The description of the patients, their date of Pandemrix vaccination, time of onset of narcolepsy and the time of serum sample collection is shown in Table S1. The diagnosis of narcolepsy (ICD-10 code G47.4) was based on the criteria of the International Classification of Sleep Disorders (version 2, ICSD-2) [4]. A Multiple Sleep Latency Test (MSLT) [1] and HLA DQB1 typing with a panel of sequence specific oligonucleotide probes [18] were carried out for all patients. Other causes of excessive daytime sleepiness (EDS) (e.g. sleep apnea, delayed sleep phase syndrome or sleep deprivation) as well as other neurological disorders (encephalitis, encephalopathy, other neurological disorders) were excluded by polysomnography, actigraphy, thorough neurological examination, magnetic resonance imaging (MRI), electroencephalography (EEG), cerebrospinal fluid (CSF) examinations, blood tests, and other examinations when necessary. Based on the review of patient records, an experienced neurologist and sleep specialist (MP, TKirjavainen), independently of each other, validated the time of onset and diagnosis of narcolepsy. If the opinion of the two specialists differed significantly, the onset time and diagnosis was verified by a panel of three neurologists/sleep specialists (alternating CH, OSH, PO, SLH).

### Serum Specimens from Narcoleptic Patients/Coupled Paired Samples/Age-matched Controls

Serum specimens from all 45 narcoleptic patients were collected during 2011. Paired serum specimens from 28 adults suffering from a clinical, laboratory-confirmed H1N1pdm09 virus infection were collected during the epidemic season 2009–2010 and 2010–2011 in the Tampere city area. H1N1pdm09 infection was confirmed by identifying viral RNA in nasopharyngeal stick samples collected during the acute phase of the disease using a diagnostic polymerase chain reaction (PCR) test with the NS gene as the PCR target [19]. Five of the patients had previously been vaccinated with Pandemrix.

Paired serum specimens were collected at the acute (at the time of the diagnosis) and convalescent phases (14–21 days later) of the disease. All patients showed a diagnostic antibody rise in the H1N1pdm09 virus-specific hemagglutination inhibition assay (HI; ≥4 × rise; Table S3) or H1N1pdm09 NS1 protein-specific Western blot assay (≥2 × rise; Table S3).

Control serum specimens from 50 randomly selected aged-matched control individuals were collected from a larger serum sample collection drawn in 2004 and 2005 (clearly prior to the 2009 pandemic) that was initially collected from diagnostic left-over serum specimens from the HUSLAB (Helsinki, Finland) diagnostic laboratory. Serum specimens were previously analysed and they were found to be negative for anti-H1N1pdm09 antibodies as analysed by means of the hemagglutination inhibition (HI) test [20] (Table S4).

### Plasmids and DNA Manipulations

Regions coding for influenza A virus non-structural protein 1 (NS1) in the cDNAs for wild-type (wt) A/Udorn/72 (H3N2) (Genebank accession number, V01102) and wt A/Finland/544/09 (H1N1pdm09) (GQ283489.1) *(NS)* genes were modified by PCR to create N- and C-terminal *Bam*HI sites for further cloning into an *E. coli* GST fusion vector pGEX-3X (Amersham Biosciences, Buckinghamshire, UK). All DNA manipulations were performed according to standard protocols, and the newly created gene constructs were fully sequenced.

### Production of Recombinant Glutathione-S-transferase (GST)-NS1 Fusion Proteins in *E. coli* BL21 Cells, and Purification of the Proteins

GST and chimeric GST-NS1 fusion proteins were expressed under isopropyl-beta-thiogalactopyranoside (IPTG) induction in *E. coli* BL21 cells. For GST and GST-NS1 protein purification, 5 mg of sonicated, cellular protein samples in Laemmli sample buffer [21] were subjected to preparative sodium dodecyl sulfate polyacrylamide gel electrophoresis (SDS-PAGE) (Model 491 Prep Cell, Bio-Rad Laboratories). The recombinant GST and GST-NS1 fusion proteins were separated on 12% and 7% gradient SDS-PAGE gels, respectively [22].

### Detection of NS1 Protein-specific Antibodies in Human Sera by Western Blotting Using *E. coli* -expressed Recombinant NS1 Proteins

To analyse humoral immune responses against NS1 proteins, serum specimens were obtained from 45 narcoleptic patients. Twenty eight paired serum samples were obtained from patients who suffered from a laboratory confirmed H1N1pdm09 virus infection, and 50 control sera were obtained from age-matched children. For Western blot analysis, 3 µg of purified GST (26 kDa) and GST-NS1 A/Udorn/72 (subtype H3N2, 52 kDa) or A/Finland/544/09 (subtype H1N1pdm09, 52 kDa) proteins were loaded on 12% SDS-PAGE gels. Proteins, separated on gels, were stained with Coomassie Brilliant Blue or transferred onto Immobilon-P membranes (polyvinylidine difluoride; Millipore) with an Isophor electrotransfer device (Hoefer Scientific Instruments, San Francisco, CA, USA). The membranes were sliced and stained with serial dilutions of tested human sera (dilutions of 1∶100, 1∶1,000, 1∶10,000, 1∶100,000) in PBS, containing 5% nonfat milk at room temperature for 2 h. After washing with PBS, secondary peroxidase-conjugated anti-human IgG immunoglobulins (Vector Laboratories, Burlingame, Inc., CA) were allowed to bind at room temperature for 1 h. After washing with PBS, the bands were visualised by 3-amino-9-ethylcarbazole (AEC) [23]. Western blot titers were determined as the last dilution showing a positive result. If the staining was exceptionally strong in lower dilution and negative in the next dilution the titer was determined to be between the dilution factors.

### Serological Hemagglutination Inhibition (HI) Test

Serum specimens, obtained from 45 narcoleptic patients, from 28 patients (56 paired serum specimens) suffering from influenza infection and 50 age-matched control individuals, were analysed by the HI test against influenza A/California/07/2009 vaccine strain and epidemic A/Finland/814/01 (H1N1) and A/Finland/715/00 (H3N2) virus strains isolated in Finland. The HI tests were performed in accordance with World Health Organization (WHO) guidelines with established procedures [24], [25] using turkey erythrocytes. Serum samples were pre-treated with receptor destroying enzyme (RDE) from Vibrio cholerae filtrate (Denka Seiken, Tokyo, Japan) at +37°C for 18 h followed by absorption with 100% turkey erythrocytes at +4°C for one hour to remove non-specific inhibitors and agglutinins. Serial 1∶2 serum dilutions, starting from 1∶10 initial dilution, were performed. Then live viral antigen (4 hemagglutinin (HA) units) was added and the samples were incubated at ambient temperature for one hour. Turkey red blood cells (0.5%) were added to antigen-serum solutions which were then incubated at room temperature for 30 minutes to determine the HI titers.

### Statistical Analysis

Anti-NS1 titers in human sera were obtained by Western blotting. These values were used for calculating the geometric mean titers with 95% confidence intervals for each patient group. The significances of the differences between the mean H1/H3 titers of narcoleptic patients and other patient groups were calculated with Student’s t-test (two tailed, unequal variance).

### Sequence Comparisons and Structural Analyses

To compare the genetic relationship of various human influenza A virus NS1 proteins the sequences for N1N1pdm09, H1N1, H2N2 and H3N2 virus NS1 genes were obtained from Genebank. The software programme Mega version 4 [26] (Molecular Evolutionary Genetics Analysis) was used for sequence comparisons of NS1 gene nucleotide and amino acid comparisons and for the construction of the phylogenetic tree. The phylogenetic tree was generated using the Neighbor-joining method [27] with the maximum composite likelihood model [28]. Bootstrapping was performed with 1000 duplicates [29].

## Results

### Pandemic Influenza A Virus Subtype H1N1pdm09 NS1 Protein Differs Considerably from the Seasonal H3N2 and H1N1 Virus NS1 Proteins

In order to study whether the patients who developed narcolepsy after Pandemrix vaccination in 2009–2010 were also suffering from an H1N1pdm09 virus infection simultaneously or after the vaccination, we carried out a computer search for potential virus proteins that could form the basis for an immunological distinction of vaccine or influenza infection-induced immunity. Since the vaccine contained the H1N1pdm09 virus (A/California/7/2009) surface glycoproteins and internal viral proteins from a vaccine backbone virus PR8 (A/Puerto Rico/34 H1N1), it is not possible to distinguish vaccine or infection-induced immunity by conventional immunological methods like the HI test, the virus neutralization test or Western blotting against viral structural proteins. However, since the Pandemrix vaccine did not contain viral NS1 protein, which is abundantly produced during virus infection, we analysed whether measuring an immune response against the NS1 protein could serve as a method to distinguish H1N1pdm09 virus infected and vaccinated individuals from those that were only vaccinated.

**Figure 1:**
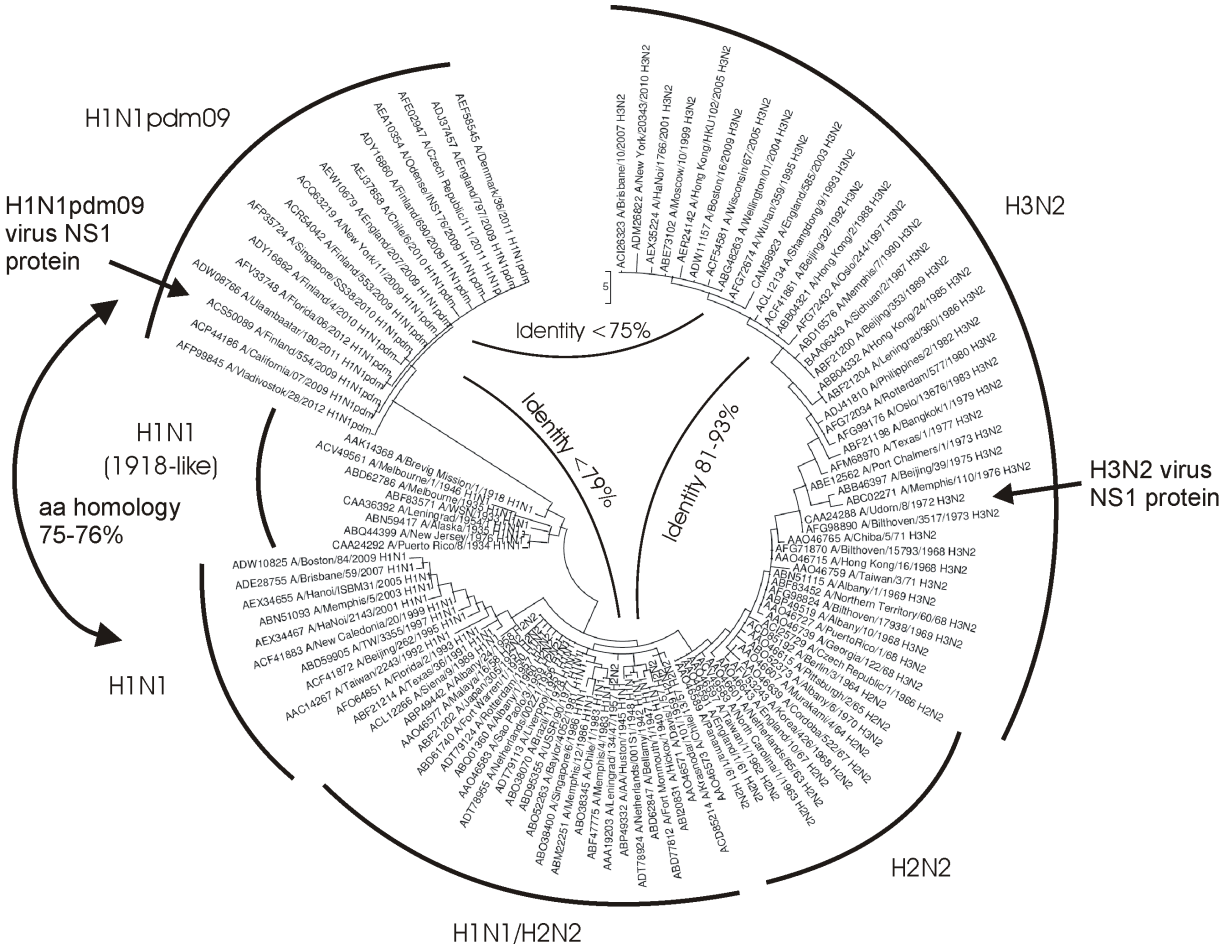
A phylogram of influenza A virus NS1 amino acid sequences from 113 human isolates representing strains circulating in human population from 1918 to 2012. The phylogram was constructed by maximum parsimony method using MEGA 3.1 software. Branch lengths represent number of amino acid changes as indicated by the scale bar. GenBank accession number and the generic name of the virus subtype are shown. Viral NS1 sequences are grouped in different subtypes as indicated in the figure.

First, we compared the amino acid homology of the NS1 proteins between the pandemic H1N1pdm09 viruses and the pandemic/seasonal H1N1, H2N2 and H3N2 viruses to evaluate the level of homology between different subtype NS1 proteins. The *NS* genes of the 20^th^ century human H1N1 and H3N2 influenza A viruses have evolved from the A/Brevig mission/18 virus [30]. The *NS* genes of the pandemic H1N1pdm viruses, however, originate from classical swine influenza viruses [31] and they are phylogenetically divergent from classical human influenza A virus *NS* genes (Figure 1). As shown in Table 1, the amino acid homology within the groups of seasonal H1N1 or H3N2 virus NS1 proteins is 88–98% and 90–100%, respectively. Also, the amino acid homology of the influenza A virus Udorn/72 NS1 (H3N2) protein, which is used in the present analysis, ranges between 84–93% and 90–97%, as compared to seasonal H1N1 and H3N2 virus NS1 proteins, respectively (Table 1). In contrast, the amino acid homology of the pandemic H1N1pdm09 NS1 protein is clearly lower, ranging between 75–79% and 73–75% as compared to H1N1 and H3N3 viruses, respectively. These differences may be sufficient to distinguish infection-induced NS1 protein-specific humoral immunity between seasonal and pandemic H1N1pdm09 influenza A virus infections.

**Table 1:** Pairwise comparisons of the NS1 sequences of selected human influenza A viruses and vaccine virus strains.

Subtype	Strain	Sequence identity (%)										
		H1N1pdm09		H1N1				H3N2				
		Fin/554/09	Cal/7/09	USSR/90/77	Sing./6/86	New Cal./20/99	Bris./59/07	Udorn/72	Bang./1/79	Sich./2/87	Mos./10/99	Bris./10/07
H1N1pdm09	Fin/554/09		**100**	**75.5**	**74.7**	**78.7**	**75.7**	**73.4**	**72.6**	**73.0**	**74.8**	**74.8**
	Cal/7/09	*100*		**75.5**	**74.7**	**78.7**	**75.7**	**73.4**	**72.6**	**73.0**	**74.8**	**74.8**
H1N1	USSR/90/77	*77.7*	*77.7*		**97.9**	**91.1**	**89.0**	**92.8**	**90.7**	**88.6**	**85.7**	**85.2**
	Sing./6/86	*77.1*	*77.1*	*98.5*		**89.9**	**88.6**	**91.6**	**88.6**	**87.3**	**84.4**	**84.0**
	New Cal./20/99	*79.6*	*79.6*	*92.0*	*91.2*		**87.8**	**85.7**	**85.2**	**83.1**	**85.2**	**85.7**
	Bris./59/07	*78.4*	*78.4*	*92.2*	*91.6*	*91.6*		**84.4**	**83.5**	**81.4**	**83.5**	**83.0**
H3N2	Udorn/72	*75.2*	*75.2*	*95.0*	*93.7*	*88.3*	*88.7*		**96.2**	**94.9**	**90.7**	**90.3**
	Bang./1/79	*75.0*	*75.0*	*93.4*	*91.9*	*87.4*	*87.4*	*97.6*		**97.0**	**92.8**	**92.4**
	Sich./2/87	*75.5*	*75.5*	*92.6*	*91.3*	*86.6*	*86.6*	*97.1*	*98.6*		**95.8**	**95.4**
	Mos./10/99	*78.1*	*78.1*	*89.4*	*88.1*	*89.0*	*89.3*	*93.0*	*94.5*	*95.9*		**99.6**
	Bris./10/07	*77.8*	*77.8*	*89.2*	*88.0*	*89.5*	*89.2*	*92.9*	*94.4*	*95.5*	*99.6*	

Figures in italic represent aligned NS1 nucleotide sequences and figures in boldface represent aligned NS1 amino acid sequences. All alignments were done using the Needleman-Wunsch algorithm and the values indicate identity percentages. Full names of the strains are as follows: A/Finland/554/2009; A/California/07/2009; A/USSR/90/77; A/Singapore/6/1986; A/New Caledonia/20/99; A/Brisbane/59/2007; A/Udorn/72; A/Bangkok/01/1979; A/Sichuan/02/87; A/Moscow/10/99; A/Brisbane/10/2007.

### Detection of NS1 Antibodies against Recombinant Influenza A Virus NS1 Proteins in Human Sera

The analysis of narcoleptic patient sera and sera obtained from H1N1pdm09 virus-infected individuals and controls was carried out using purified recombinant influenza A/Udorn/72 (H3N2) and influenza A/Finland/554/09 (H1N1pdm09) NS1 proteins by Western blot technique. Western blot analysis was used since the purification of NS1 protein in a native form is very difficult due to its insolubility and stickiness. In addition, we had previously good experience in analyzing specific antibody responses against recombinant microbial proteins by Western blotting [32], [33]. The method is highly specific and unlikely vulnerable to nonspecific binding to impurities in antigen preparation seen e.g. in the enzyme immune assay methods.

**Figure 2:**
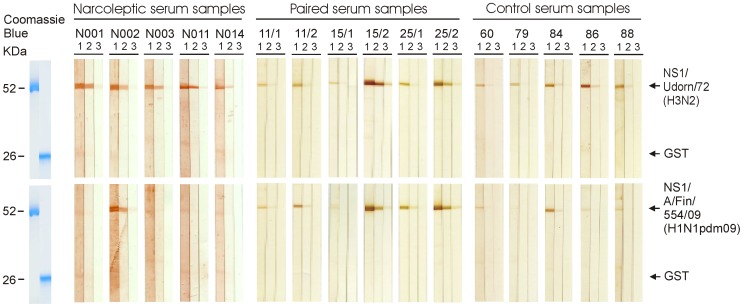
Analysis of NS1 protein-specific antibody responses in human sera by using recombinant influenza A virus NS1 proteins. Three µg of *E. coli*-expressed and preparative SDS-PAGE -purified recombinant proteins was loaded onto 12% SDS-PAGE gels. GST (26 kDa) and GST-NS1 A/Udorn/72 (H3N2) (52 kDa) proteins were loaded onto one gel and GST and GST-NS1 A/Finland/544/09 (H1N1) (52 kDa) proteins onto another gel, respectively. Proteins separated on gels were transferred onto nylon membranes. The membranes were sliced and stained with serially diluted human sera, obtained from five narcoleptic patients (patients: N001, N002, N003, N011, N014). In addition, paired serum samples were obtained from three patients, who had suffered from a laboratory confirmed H1N1pdm09 virus infection (patients: 11/1;acute phase serum sample from patient 11), 11/2 (convalescent sample from patient 11), 15/1, 15/2, 25/1, 25/2), and five serum samples were obtained from age-matched, control individuals (samples: 60, 79, 84, 86, 88), as indicated in the figure. The following dilutions were used: 1∶100 (lane 1), 1∶1,000 (lane 2) and 1∶10,000 (lane 3). After incubation with secondary Abs, the bands were visualized by staining with 3-amino-9-ethylcarbazole (AEC). GST and GST-NS1 proteins were visualized by staining with Coomassie Brilliant Blue as shown on the left.

Serum specimens showed antibody reactivity against influenza A virus NS1 proteins (GST-NS1 fusion protein) but hardly any reactivity against the GST protein (Figure 2). All tested serum specimens were positive for the H3N2 virus (Udorn/72) NS1 protein, while the reactivity against the H1N1pdm09 NS1 protein was equal to or clearly lower than that of the Udorn NS1 protein reactivity.

### Sera from Narcoleptic Children and Adolescent Show High Antibody Reactivity to the H3N2 Subtype Influenza A Virus NS1 Protein but Low Reactivity to the H1N1pdm09 Subtype Influenza A Virus NS1 Protein

**Figure 3:**
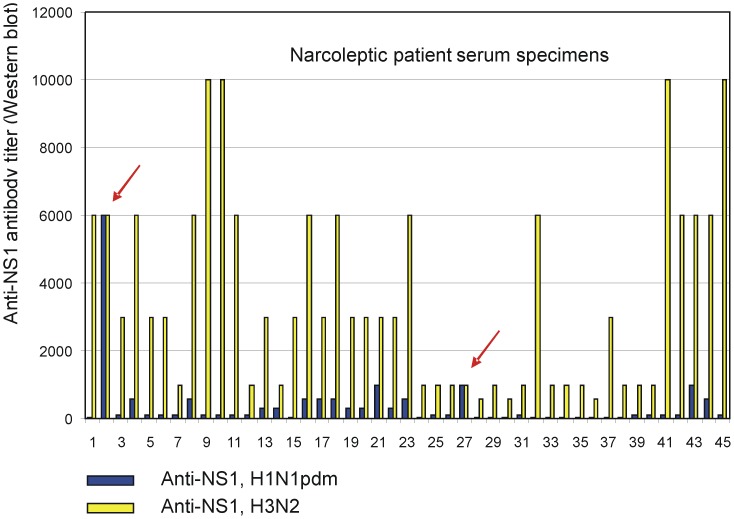
Column charts showing antibody titers against H1N1pdm09 subtype and H3N2 subtype influenza A virus NS1 proteins in serum specimens obtained from narcoleptic patients. The relative antibody levels against N1N1pdm09 and H3N2 virus NS1 proteins were determined as the last serum dilution showing a positive signal in Western blot analysis. Blue columns show antibody titers against H1N1pdm09 subtype, and yellow columns show antibody titers against H3N2 subtype influenza A virus NS1 proteins in serum specimens obtained from 45 narcoleptic patients. Sera 2 and 27 (red arrows) show equally high titer levels against both NS1 protein subtypes.

Antibody titers against H1N1pdm09 subtype and H3N2 subtype influenza A virus NS1 proteins in serum specimens were analysed from 45 narcoleptic patients (Figure 3, Tables S1, S2). The NS1 protein proved to be highly immunogenic, since all serum specimens were positive and showed high or relatively high antibody levels against the H3N2 subtype influenza A virus NS1 protein. The serum reactivity against the H1N1pdm09 subtype NS1 protein was generally very low and there were only two individuals (Fig. 3, cases 2 and 27) whose anti-H1N1pdm09 NS1 protein-specific antibodies were equal to those seen against the H3N2 virus NS1 protein. Antibody titers against H1N1pdm09 subtype and H3N2 subtype NS1 proteins ranged from <100 (detection level was 1∶100 of serum dilution) to 6000 and from 600 to 10,000, respectively. Geometric mean antibody titers against H1N1pdm09 subtype and H3N2 subtype NS1 proteins in serum specimens were 156 (95% CI, 111.4–218.6) and 2502 (95% CI, 1921.0–3257.9), respectively (Table 2).

**Table 2:** Statistical analyses of serum specimens for each patient group.

Serum samples;		n	Geom. mean		95% confidence interval	
anti-NS1						
Narcoleptic patients	H1pdm09	45	156.1		111.4−218.6	
Control patients	H1pdm09	50	418.2		300.3−582.4	
Acute phase patient sera	H1pdm09	28	551.1		285.6−1063.4	
Convalescent phase patient sera	H1pdm09	28	8413.6		4294.9−16482.1	
Narcoleptic patients	H3	45	2501.7		1921.0−3257.9	
Control patients	H3	50	1637.1		1313.6−2040.3	
Acute phase patient sera	H3	28	1218.9		721.6−2058.8	
Convalescent phase patient sera	H3	28	8536.3		4454.4−16359.0	
**Serum samples;**		**Serum samples;**				**p-value**
**anti-NS1**		**anti-NS1**				
Narcoleptic patients H1pdm	vs.	Control patients		H1pdm		<0.001
	vs.	Acute phase patient sera		H1pdm		<0.002
	vs.	Convalescent phase patient sera		H1pdm		<0.001
Narcoleptic patients H3	vs.	Control patients		H3		0.018
	vs.	Acute phase patient sera		H3		0.021
	vs.	Convalescent phase patient sera		H3		<0.002

Geometric mean antibody titers and 95% confidence interval against H1N1pdm09 subtype and H3N2 subtype NS1 proteins in serum specimens for each patient group.

Significance of the differences between the mean anti-NS1 H1/H3 virus protein antibody titers of narcoleptic patients and other patient groups.

Significance of the differences was calculated with Student’s t-test (two tailed, unequal variance).

### Paired Serum Specimens from H1N1pdm09 Virus-infected Patients Showed High Levels or a Diagnostic Rise in Antibody Titers against the H1N1pdm09 Subtype Influenza A Virus NS1 Protein and High Cross-reactivity with the H3N2 Subtype NS1 Protein

**Figure 4:**
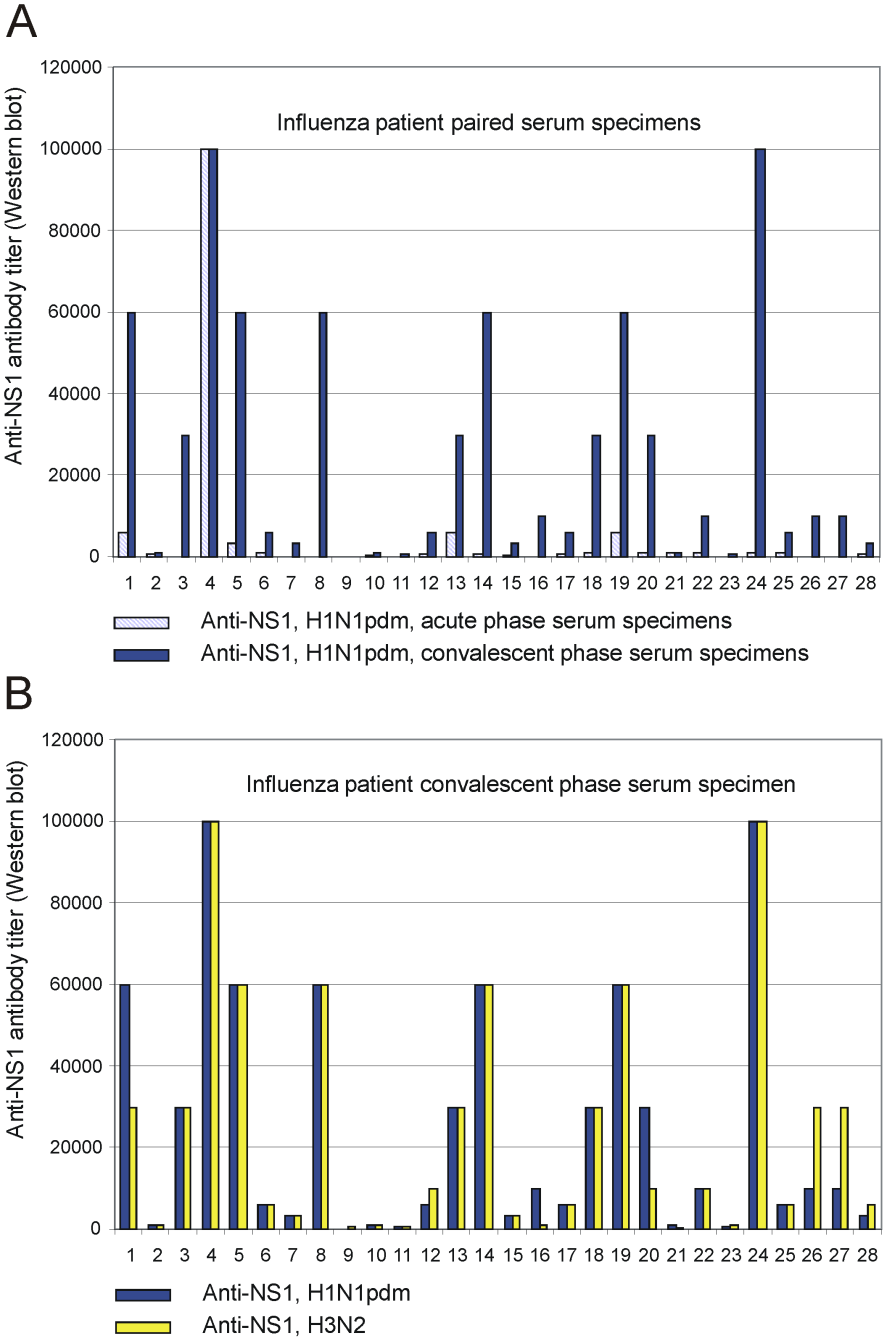
Column charts showing acute and convalescent serum antibody titers against H1N1pdm09 subtype influenza A virus NS1 protein in serum specimens obtained from 28 patients who had suffered from pandemic H1N1pdm09 virus infection. A) Striped, light blue columns show antibody titers against H1N1pdm09 subtype NS1 protein from acute phase serum specimens, and blue columns show corresponding antibody titers from convalescent serum specimens in paired serum samples obtained from 28 patients who had suffered from H1N1pdm09 influenza virus infection. All 28 patients had a laboratory confirmed infection as verified by positive results with viral M and NS RNA-positive RT-PCR test. B) Blue columns show antibody titers against H1N1pdm09 subtype NS1 protein from convalescent serum specimens, and yellow columns show antibody titers against H3N2 subtype NS1 protein from the same serum specimens obtained from 28 laboratory confirmed influenza infection patients.

In order to analyse whether clinical H1N1pdm09 infection leads to the induction of NS1 protein-specific antibodies we analysed paired serum samples from 28 laboratory confirmed influenza A virus-infected patients. The samples had been collected during the 1^st^ and 2^nd^ pandemic season in 2009/2010 and 2010/2011, when the epidemic influenza A virus was almost solely of the H1N1pdm09 subtype. Almost all (89%) patients who had suffered from an H1N1pdm09 virus infection showed high or very high levels (>600) or a significant rise (>2 x) in antibody titers against the H1N1pdm09 subtype influenza A virus NS1 protein (Figure 4A, Table S3). It is noteworthy that the H1N1pdm09 virus infection also induced NS1-specific responses that were strongly cross-reactivity with the H3N2 subtype virus NS1 protein (Figure 4B, Table S3). The rise in geometric mean titers was 15-fold, from 551 (95% CI, 285.6–1063.4) to 8414 (95% CI, 4294.9–16482.1), against the H1N1pdm09 subtype NS1 protein, and 7-fold against the H3N2 subtype NS1 protein (from 1219 (95% CI, 721,6–2058,8) to 8536 (95% CI, 4454,4–16359,0)), respectively (Table 2).

### Control Sera from 50 Age-matched Children and Adolescent Showed High Antibody Reactivity against the H3N2 Subtype Influenza A Virus NS1 Protein but Low Cross-reactivity to the H1N1pdm09 Subtype NS1 Protein

As an additional control, the antibody titers against the H1N1pdm09 subtype and the H3N2 subtype influenza A virus NS1 proteins were determined in serum specimens collected from 50 age-matched control children and adolescent (Table S4). The serum specimens had been collected during the years 2004 and 2005, several years before the appearance of the pandemic H1N1pdm09 virus. Also among these individuals NS1 protein appeared to be highly immunogenic and all sera had antibodies against the H3N2 subtype NS1 protein. Antibody titers against H1N1pdm09 subtype and H3N2 subtype NS1 proteins ranged from <100 to 6000 and from 300 to 6000, respectively (Table S4). Geometric mean antibody titers against H1N1pdm09 subtype and H3N2 subtype NS1 proteins in serum specimens were 418 (95% CI, 300.3–582.4) and 1637 (95% CI, 1313.6–2040.3), respectively (Table 2). All specimens were negative for the pandemic H1N1pdm09 virus as analysed by the HI test (Table S4). Among these children the H3N2 infection had been far more frequent than the seasonal H1N1 infection, since the seropositivity rate and the GMT levels against seasonal H3N2 virus (86% seropositivity, GMT 86) were clearly higher as compared to that of a seasonal H1N1 virus (54% seropositivity, GMT 15) (Table S4).

A summary of anti-NS1 protein-specific GMTs and statistical differences between the groups is shown in Table 2. Serum specimens from narcoleptic patients showed highly significantly lower anti-H1N1pdm09 NS1 protein antibody levels as compared to those seen in virus-infected patients and control individuals (Table 2). However, such differences were not observed in anti-H3N2 NS1 protein-specific responses, with the exception of convalescent serum specimens from H1N1pdm09 virus-infected patients, who showed significant induction of cross-reactive antibodies recognising also H3N2 virus NS1 protein (Table 2).

## Discussion

Narcolepsy is considered as an immune-related disease, but the precise mechanism of disease pathogenesis and etiology has remained unresolved. Surprisingly, in 2010 in Sweden and Finland it was observed that the incidence of childhood and adolescent narcolepsy suddenly increased [14], [15], [16], [34]. In both countries this increase was observed several months after the pandemic that peaked in October-November 2009 and the national Pandemrix vaccination campaigns that took place between October 2009 and February 2010. Increased incidence of narcolepsy was subsequently observed in other European countries as well [17]. The observations of the increased risk of narcolepsy in vaccinated children and adolescents in comparison to unvaccinated age-matched subjects suggested that AS03-adjuvanted Pandemrix vaccination, perhaps together with other environmental factors, contributed to the increased incidence of narcolepsy in children in Finland in 2010 [15]. As the onset of narcolepsy has been shown to be seasonal and the cases of narcolepsy have increased following the 2009 H1N1 pandemic in China [11], we wanted to analyse whether H1N1pdm09 virus infection, together with the AS03-adjuvanted Pandemrix vaccination, contributed to the abrupt increase in the incidence of childhood narcolepsy in Finland.

Han and co-workers found a 3-fold higher incidence of narcolepsy following the 2009 H1N1pdm09 virus pandemic, which appeared to be unrelated to influenza vaccination [11]. This increased incidence of childhood narcolepsy decreased to baseline within two years after the 2009 H1N1 pandemic, indicating that the exceptionally high incidence of narcolepsy occurred only after the winter of 2009–2010 [35]. Nohynek and co-workers found no evidence of a change in the incidence of narcolepsy among unvaccinated 4–19-year-olds after the first H1N1pdm09 epidemic year in Finland, whereas a considerably increased (13-fold) risk was associated with Pandemrix vaccination [15]. In the present study, children/adolescents suffering from narcolepsy showed high antibody levels to the H3N2 subtype influenza A virus NS1 protein but very low antibody levels to the H1N1pdm09 subtype NS1 protein (Figures 2, 3, Table 2, Table S2). This was also the case with age-matched control sera collected in 2004 and 2005 (Figure 2, Table 2, Table S3). Instead, patients who had suffered from a pandemic H1N1pdm09 influenza virus infection showed high antibody levels against the H1N1pdm09 subtype NS1 protein and equally high cross-reactivity to the H3N2 subtype influenza A virus NS1 protein (Figures 2, 4A, B, Table 2, Table S3). These results strongly suggest that pandemic H1N1pdm09 influenza virus infection was not the main contributing factor for the increased incidence of narcolepsy in Finland in the beginning of 2010. In our analysis only 2 out of 45 narcoleptic patient sera (4.4%) showed equally high antibody levels against H1N1pdm09 and H3N2 virus NS1 proteins, which provide indirect evidence that H1N1pdm09 virus infection was not common among our narcoleptic patients. In addition, clinical data from our patients indicated that influenza-like illness (ILI) was found only in a minority of our patients and other symptomatic upper respiratory tract infection such as sore throat (streptococcal infection) were not observed at all [14]. Our data, however, do not rule out the possibility that infections such as influenza or streptococcal infections could contribute to the onset of narcolepsy in children and adolescent at the population level, but we did not find any evidence of infections playing a significant role in our narcolepsy patients. It also has to be pointed out that the nationwide Infectious Disease Registry, which collects data on microbial infections in Finland does not provide evidence that any of the 15 000 confirmed H1N1pdm09 infection cases (all ages) diagnosed in 2009–2011 have developed narcolepsy.

Our findings [14], [15] are supported by studies from Sweden and U.K., which reported significant increase in the incidence of narcolepsy with cataplexy in children vaccinated with Pandemrix as compared to those in the same age group who were not vaccinated [16], [17], [34]. Preliminary data from France, Norway and Ireland also indicate a higher than expected number of narcolepsy cases in children and adolescents after Pandemrix vaccination [36], [37], [38], suggesting that in genetically susceptible children Pandemrix vaccination may alone be sufficient to initiate/accelerate narcolepsy.

The strongest genetic risk factor for narcolepsy is associated with a specific HLA class II gene [9], [10]. More than 90% of patients with a cataplexic form of narcolepsy carry the HLA DQB1*06:02 allele compared to a 27% frequency of the allele in the general Finnish population. In the present study, all our patients were positive for the HLA DQB1*06:02 allele showing a very strong correlation of narcolepsy with this risk allele. Because antibodies to NS1 protein were seen in 96% of controls with confirmed H1N1 infection and about 27% have the HLA DQB1*06:02 allele, the presence of HLA DQB1*06:02 allele in the patients with narcolepsy seems not to be the explanation for the lack of antibody response to the NS1 protein. Furthermore, antibody responses to the H3 NS1 protein were seen in all our narcolepsy patients.

Altogether, in Finland approximately 600 000 children and adolescent were vaccinated with Pandemrix vaccine in 2009–2010 giving a theoretical figure of 150 000 children and teens with a positive HLA DQB1*06:02 susceptibility gene to be at risk for developing narcolepsy. At present, approximately 120 individuals aged 5–18 have developed narcolepsy after the Pandemrix vaccination campaign. This gives a prevalence figure of 80/100 000 vaccinated HLA DQB1*06:02 positive children. The figure is high, but it also suggests that additional genetic and environmental predisposing factors likely contribute to the onset of Pandemrix-associated narcolepsy. At present, we are not aware of other obvious predisposing factors apart from Pandemrix vaccination.

Presently, we do not know the mechanism by which the viral antigen or the components of the AS03 adjuvant (squalene, polysorbate80 or DL-alpha-tocopherol), and/or different excipients included in the suspensions could contribute to the induction or enhancement of autoimmunity, leading to the onset of symptomatic narcolepsy. Theoretically, it is possible that any pro-inflammatory signal, whether it originates from a microbial infection or vaccination, such as inflammation inducing Pandemrix vaccine, could trigger further development of autoimmunity by a non-specific “bystander” mechanism, leading to clinical onset of narcolepsy with cataplexy. We did not, however, find evidence for influenza infection as a significant contributing factor in the etiology of narcolepsy in our patients.

## Supporting Information

Table S1 **Clinical and laboratory data of 45 narcoleptic patients.**
(DOC)

Table S2 **Anti-NS1 and virus-specific hemagglutination inhibition (HI) titers from 45 narcoleptic patients.**
(DOC)

Table S3 **Anti-NS1 and virus-specific hemagglutination inhibition (HI) titers from 28 paired serum samples.**
(DOC)

Table S4 **Anti-NS1 and virus-specific hemagglutination inhibition (HI) titers from 50 age-matched controls.**
(DOC)
