# Supplementary material for: No Serological Evidence of Influenza A H1N1pdm09 Virus Infection as a Contributing Factor in Childhood Narcolepsy after Pandemrix Vaccination Campaign in Finland
Source: PLoS One. 2013 Aug 8;8(8):e68402. doi: 10.1371/journal.pone.0068402 (PMC3738560; doi:10.1371/journal.pone.0068402)
Supplement: Table S1 — Clinical and laboratory data of 45 narcoleptic patients. (DOC) [file pone.0068402.s001.doc]

| **Table S1.** Data from 45 narcoleptic patients. | | | | | |
| --- | --- | --- | --- | --- | --- |
| Case N=45 | HLA DQB *06:02 | Year of birth | Pandemri vaccinatio (Date) | Onset of symptoms after vaccinatio (Days) | Sampling time after vaccinatio (Days) |
| N001 | + | 2002 | 12/2/2009 | 98 | 404 |
| N002 | + | 1997 | 12/10/2009 | 67 | 398 |
| N003 | + | 1999 | 11/1/2009 | 83 | 428 |
| N004 | + | 1995 | 11/11/2009 | 39 | 427 |
| N005 | + | 1998 | 11/27/2009 | 0 | 417 |
| N006 | + | 1998 | 11/25/2009 | 110 | 420 |
| N007 | + | 1993 | 12/14/2009 | 32 | 413 |
| N008 | + | 2001 | 11/16/2009 | 0 | 444 |
| N009 | + | 1996 | 11/20/2009 | 25 | 445 |
| N010 | + | 1999 | 11/16/2009 | 45 | 449 |
| N011 | + | 1996 | 11/10/2009 | 83 | 456 |
| N013 | + | 1998 | 12/1/2009 | 31 | 463 |
| N014 | + | 1998 | 11/26/2009 | 11 | 468 |
| N015 | + | 1998 | 11/26/2009 | 105 | 473 |
| N021 | + | 2001 | 11/24/2009 | 75 | 490 |
| N024 | + | 1997 | 11/27/2009 | 35 | 521 |
| N025 | + | 2000 | 11/30/2009 | 20 | 525 |
| N026 | + | 1994 | 12/1/2009 | 31 | 534 |
| N027 | + | 1999 | 1/8/2009 | 19 | 538 |
| N028 | + | 1998 | 11/26/2009 | 50 | 550 |
| N029 | + | 2002 | 11/26/2009 | 28 | 551 |
| N030 | + | 1995 | 12/1/2009 | 197 | 546 |
| N031 | + | 1996 | 12/9/2009 | 96 | 544 |
| N033 | + | 2001 | 11/26/2009 | 0 | 557 |
| N035 | + | 1995 | 12/3/2009 | 133 | 552 |
| N038 | + | 1999 | 12/4/2009 | 28 | 552 |
| N039 | + | 1995 | 12/4/2009 | 28 | 551 |
| N040 | + | 1997 | 121/2009 | 104 | 556 |
| N042 | + | 1998 | 12/14/2009 | 63 | 546 |
| N043 | + | 2001 | 11/23/2009 | 37 | 567 |
| N044 | + | 2005 | 11/19/2009 | 57 | 572 |
| N047 | + | 2002 | 11/21/2009 | 24 | 583 |
| N048 | + | 1995 | 11/9/2009 | 248 | 596 |
| N049 | + | 1995 | 12/10/2009 | 36 | 566 |
| N050 | + | 1998 | 12/15/2009 | 62 | 561 |
| N051 | + | 1998 | 11/16/2009 | 29 | 591 |
| N052 | + | 1997 | 11/4/2009 | 56 | 603 |
| N053 | + | 2000 | 11/24/2009 | 111 | 582 |
| N056 | + | 2003 | 2/10/2010 | 186 | 531 |
| N060 | + | 1996 | 11/26/2009 | 262 | 629 |
| N061 | + | 1999 | 11/24/2009 | 264 | 643 |
| N062 | + | 2000 | 11/14/2009 | 40 | 653 |
| N063 | + | 1997 | 12/1/2009 | 207 | 639 |
| N064 | + | 1994 | 12/3/2009 | 133 | 643 |
| N069 | + | 2003 | 11/23/2009 | 555 | 728 |
| HLA; human leukocyte antigen | | | | | |
