# Supplementary material for: No Serological Evidence of Influenza A H1N1pdm09 Virus Infection as a Contributing Factor in Childhood Narcolepsy after Pandemrix Vaccination Campaign in Finland
Source: PLoS One. 2013 Aug 8;8(8):e68402. doi: 10.1371/journal.pone.0068402 (PMC3738560; doi:10.1371/journal.pone.0068402)
Supplement: Table S2 — Anti-NS1 and virus-specific hemagglutination inhibition (HI) titers from 45 narcoleptic patients. (DOC) [file pone.0068402.s002.doc]

| **Table S2.** Anti-NS1 and hemagglutination inhibition (HI) specific antibody titers in 45 narcoleptic patient serum specimens. | | | | | |
| --- | --- | --- | --- | --- | --- |
|  | **Anti-NS1** | **Anti-NS1** | **Anti-virus** | **Anti-virus** | **Anti-virus** |
| Case N=45 | A/Finland 544/09 H1N1pd WB titers | A/Udorn/72 H3N2 | A/California/7/09 H1N1pdm | A/Finland 814/01 H1N1 HI titers | A/finland/ 715/00 H3N2 HI titers |
|  |  |  |  |  |  |
|  |  | WB titers | HI titers |  |  |
| N001 | <100 | 6000 | 160 | 80 | <10 |
| N002 | 6000 | 6000 | 320 | <10 | 320 |
| N003 | 100 | 3000 | 40 | 160 | 40 |
| N004 | 600 | 6000 | 320 | 10 | 5120 |
| N005 | 100 | 3000 | 160 | 20 | 320 |
| N006 | 100 | 3000 | 20 | 10 | 160 |
| N007 | 100 | 1000 | 80 | <10 | 2560 |
| N008 | 600 | 6000 | 320 | 80 | 1280 |
| N009 | 100 | 10000 | 80 | 320 | 10 |
| N010 | 100 | 10000 | 20 | 80 | <10 |
| N011 | 100 | 6000 | 80 | 40 | 320 |
| N013 | 100 | 1000 | 320 | 80 | 5120 |
| N014 | 300 | 3000 | 80 | 320 | <10 |
| N015 | 300 | 1000 | 80 | 160 | 1280 |
| N021 | <100 | 3000 | 40 | 20 | <10 |
| N024 | 600 | 6000 | 80 | 640 | 2560 |
| N025 | 600 | 3000 | 1280 | 1280 | 10 |
| N026 | 600 | 6000 | 1280 | 80 | 2560 |
| N027 | 300 | 3000 | 40 | 160 | 20 |
| N028 | 300 | 3000 | 80 | 40 | 640 |
| N029 | 1000 | 3000 | 10240 | 40 | 10 |
| N030 | 300 | 3000 | 80 | 160 | 2560 |
| N031 | 600 | 6000 | 320 | 40 | 320 |
| N033 | <100 | 1000 | 20 | 640 | <10 |
| N035 | 100 | 1000 | 1280 | 10 | 2560 |
| N038 | 100 | 1000 | 40 | 160 | 320 |
| N039 | 1000 | 1000 | 160 | 20 | 40 |
| N040 | <100 | 600 | 10240 | 160 | 10240 |
| N042 | <100 | 1000 | 40 | 160 | 320 |
| N043 | <100 | 600 | 20 | 10 | 40 |
| N044 | 100 | 1000 | 40 | 40 | <10 |
| N047 | <100 | 6000 | 40 | 40 | 10 |
| N048 | <100 | 1000 | 20 | 80 | 640 |
| N049 | <100 | 1000 | 20 | 40 | 10 |
| N050 | <100 | 1000 | 80 | 10 | 320 |
| N051 | <100 | 600 | 160 | 80 | <10 |
| N052 | <100 | 3000 | 2560 | 40 | 10240 |
| N053 | <100 | 1000 | 40 | 40 | 320 |
| N056 | 100 | 1000 | 640 | <10 | <10 |
| N060 | 100 | 1000 | 320 | 40 | 640 |
| N061 | 100 | 10000 | 20 | 40 | 20 |
| N062 | 100 | 6000 | 20 | 160 | 40 |
| N063 | 1000 | 6000 | 80 | 320 | 1280 |
| N064 | 600 | 6000 | 5120 | 40 | 2560 |
| N069 | 100 | 10000 | 40 | 80 | 10 |
| **GMT:** | 154.6 | 2501.7 | 129.0 | 57.9 | 139.3 |
| WB; Western blot, HI; hemagglutination inhibition, GMT; geometric mean titer | | | | | |
