# Supplementary material for: No Serological Evidence of Influenza A H1N1pdm09 Virus Infection as a Contributing Factor in Childhood Narcolepsy after Pandemrix Vaccination Campaign in Finland
Source: PLoS One. 2013 Aug 8;8(8):e68402. doi: 10.1371/journal.pone.0068402 (PMC3738560; doi:10.1371/journal.pone.0068402)
Supplement: Table S3 — Anti-NS1 and virus-specific hemagglutination inhibition (HI) titers from 28 paired serum samples. (DOC) [file pone.0068402.s003.doc]

| **Table S3.** Anti-NS1 and hemagglutination inhibition-specific antibody titers in 28 paired serum samples of influenza patients*. | | | | | | | | | | |
| --- | --- | --- | --- | --- | --- | --- | --- | --- | --- | --- |
| Case N=28 | **Anti-NS1** | | **Anti-NS1** | | **Anti-virus** | | **Anti-virus** | | **Anti-virus** | |
|  | A/Finland/544/09 | | A/Udorn/72 | | A/California/7/09 | | A/Finland/814/01 | | A/Finland/715/00 | |
|  | H1N1pdm | | H3N2 | | H1N1pdm | | H1N1 | | H3N2 | |
|  | WB titers | | WB titers | | HI titers | | HI titers | | HI titers | |
|  | I | II | I | II | I | II | I | II | I | II |
| 1 | 6000 | 60000 | 3000 | 30000 | <10 | 10 | <10 | <10 | 320 | 320 |
| 2 | 600 | 1000 | 600 | 1000 | <10 | 20 | <10 | <10 | 80 | 80 |
| 3 | 100 | 30000 | 1000 | 30000 | <10 | 80 | <10 | 10 | 10 | 20 |
| 4 | 100000 | 100000 | 100000 | 100000 | 10 | 40 | 10 | 40 | 40 | 80 |
| 5 | 3000 | 60000 | 3000 | 60000 | <10 | <10 | <10 | <10 | 10 | 10 |
| 6 | 1000 | 6000 | 3000 | 6000 | 40 | 5120 | <10 | <10 | 160 | 160 |
| 7 | 100 | 3000 | <100 | 3000 | <10 | 640 | <10 | 320 | 10 | 10 |
| 8 | <100 | 60000 | 1000 | 60000 | <10 | 10 | 80 | 80 | 20 | 20 |
| 9 | 100 | 100 | 600 | 600 | <10 | 10240 | 20 | 160 | 80 | 640 |
| 10 | 300 | 1000 | 600 | 1000 | 10 | 40 | 320 | 320 | 1280 | 2560 |
| 11 | <100 | 600 | 300 | 600 | <10 | 40 | 80 | 80 | 80 | 40 |
| 12 | 600 | 6000 | 1000 | 10000 | <10 | 160 | 20 | 80 | 2560 | 2560 |
| 13 | 6000 | 30000 | 10000 | 30000 | <10 | 160 | 80 | 320 | 20 | 40 |
| 14 | 600 | 60000 | 1000 | 60000 | <10 | 1280 | 2560 | 2560 | 5120 | 5120 |
| 15 | 300 | 3000 | 1000 | 3000 | 10 | 2560 | <10 | 320 | 640 | 1280 |
| 16 | 100 | 10000 | 600 | 1000 | <10 | 160 | 20 | 20 | 5120 | 1280 |
| 17 | 600 | 6000 | 1000 | 6000 | 80 | 10240 | 20 | 10 | 160 | 320 |
| 18 | 1000 | 30000 | 3000 | 30000 | 40 | 160 | 320 | 320 | 160 | 80 |
| 19 | 6000 | 60000 | 6000 | 60000 | <10 | 80 | 10 | 40 | 160 | 320 |
| 20 | 1000 | 30000 | 1000 | 10000 | <10 | 160 | 640 | 640 | 2560 | 640 |
| 21 | 1000 | 1000 | 300 | 300 | 640 | 10240 | <10 | <10 | <10 | <10 |
| 22 | 1000 | 10000 | 3000 | 10000 | <10 | 20 | <10 | 20 | 20 | 20 |
| 23 | <100 | 600 | 100 | 1000 | <10 | 20 | <10 | 20 | 20 | 40 |
| 24 | 1000 | 100000 | 3000 | 100000 | <10 | 40 | 40 | 80 | 10 | 20 |
| 25 | 1000 | 6000 | 1000 | 6000 | <10 | 320 | <10 | <10 | 80 | 80 |
| 26 | 100 | 10000 | 1000 | 30000 | <10 | 10 | <10 | <10 | <10 | <10 |
| 27 | 100 | 10000 | 1000 | 30000 | <10 | <10 | 640 | 1280 | 40 | 40 |
| 28 | 600 | 3000 | 1000 | 6000 | <10 | 2560 | <10 | 20 | 20 | 20 |
| **GMT:** | 551.1 | 8413.6 | 1218.9 | 8536.3 | 8.2 | 144.9 | 22.1 | 47.6 | 86.2 | 100.0 |
| *All patients had an RT-PCR-confirmed H1N1pdm09 virus infection with influenza-like illness. WB, Western blot, HI; hemagglutination inhibition, I; acute phase patient sera, II; convalescent phase patient sera, GMT; geometric mean titer | | | | | | | | | | |
