# Supplementary material for: No Serological Evidence of Influenza A H1N1pdm09 Virus Infection as a Contributing Factor in Childhood Narcolepsy after Pandemrix Vaccination Campaign in Finland
Source: PLoS One. 2013 Aug 8;8(8):e68402. doi: 10.1371/journal.pone.0068402 (PMC3738560; doi:10.1371/journal.pone.0068402)
Supplement: Table S4 — Anti-NS1 and virus-specific hemagglutination inhibition (HI) titers from 50 age-matched controls. (DOC) [file pone.0068402.s004.doc]

| **Table S4.** Anti-NS1 and hemagglutination inhibition-specific antibody titers in serum specimens from 50 age-matched controls. | | | | | |
| --- | --- | --- | --- | --- | --- |
| Case N=50 | **Anti-NS1** A/Finland 544/09 H1N1pd | **Anti-NS1** A/Udorn/72 H3N2 | **Anti-virus** A/Finland 544/09 H1N1pd | **Anti-virus** A/Finland 814/01 H1N1 | **Anti-virus** A/Finland 715/00 H3N2 |
|  | WB titers | WB titers | HI titers | HI titers | HI titers |
| 1 | 300 | 1000 | <10 | 80 | 40 |
| 2 | 100 | 600 | <10 | 10 | 80 |
| 3 | 100 | 600 | <10 | 20 | 40 |
| 4 | <100 | 600 | <10 | <10 | 160 |
| 5 | 1000 | 6000 | <10 | <10 | <10 |
| 6 | <100 | 300 | <10 | 40 | 160 |
| 7 | 600 | 1000 | <10 | <10 | 80 |
| 8 | 300 | 3000 | <10 | <10 | 40 |
| 9 | 100 | 3000 | <10 | 160 | 160 |
| 10 | 1000 | 6000 | <10 | 160 | 320 |
| 11 | 600 | 3000 | <10 | <10 | <10 |
| 12 | 600 | 3000 | <10 | 10 | 40 |
| 13 | 600 | 6000 | <10 | 80 | 320 |
| 14 | 1000 | 3000 | <10 | 40 | <10 |
| 15 | 3000 | 6000 | <10 | 80 | 40 |
| 16 | 1000 | 3000 | <10 | 40 | 80 |
| 17 | 600 | 3000 | <10 | <10 | <10 |
| 18 | 100 | 6000 | <10 | 40 | 40 |
| 19 | 600 | 3000 | <10 | 20 | 40 |
| 20 | 300 | 1000 | <10 | <10 | 40 |
| 21 | 600 | 3000 | <10 | <10 | 320 |
| 22 | 300 | 1000 | <10 | <10 | 320 |
| 23 | 600 | 1000 | <10 | <10 | <10 |
| 24 | 300 | 1000 | <10 | <10 | 20 |
| 25 | 300 | 1000 | <10 | <10 | 320 |
| 26 | 300 | 1000 | <10 | 20 | 320 |
| 27 | 300 | 600 | <10 | <10 | 80 |
| 28 | 600 | 1000 | <10 | <10 | 320 |
| 29 | 100 | 1000 | <10 | 40 | 40 |
| 30 | 100 | 1000 | <10 | 40 | <10 |
| 31 | 100 | 1000 | <10 | <10 | 40 |
| 32 | 300 | 1000 | <10 | 40 | 640 |
| 33 | 100 | 3000 | <10 | <10 | 40 |
| 34 | 6000 | 6000 | <10 | 20 | 2560 |
| 35 | 3000 | 1000 | <10 | 20 | 160 |
| 36 | 600 | 3000 | <10 | <10 | 1280 |
| 37 | 100 | 1000 | <10 | <10 | 160 |
| 38 | 100 | 3000 | <10 | <10 | 320 |
| 39 | <100 | 600 | <10 | <10 | 160 |
| 40 | 1000 | 1000 | <10 | 20 | 160 |
| 41 | 300 | 600 | <10 | 40 | 320 |
| 42 | 1000 | 3000 | <10 | 80 | 160 |
| 43 | 600 | 3000 | <10 | 40 | 320 |
| 44 | 1000 | 3000 | <10 | 40 | 640 |
| 45 | 300 | 1000 | <10 | <10 | 320 |
| 46 | 3000 | 3000 | <10 | 20 | 80 |
| 47 | 3000 | 1000 | <10 | 40 | 80 |
| 48 | 3000 | 3000 | <10 | <10 | <10 |
| 49 | 1000 | 1000 | <10 | <10 | 160 |
| 50 | 1000 | 600 | <10 | 20 | 80 |
| **GMT:** | 418.2 | 1637.1 | 5.0 | 14.5 | 85.7 |
| WB; Western blot, HI; hemagglutination inhibition, GMT; geometric mean titer | | | | | |
